# Spatio-temporal variation in oxidative status regulation in a small mammal

**DOI:** 10.7717/peerj.7801

**Published:** 2019-10-08

**Authors:** Vincent Lemieux, Dany Garant, Denis Reale, Patrick Bergeron

**Affiliations:** 1 Départment de biologie, Université de Sherbrooke, Sherbrooke, Canada; 2 Biological Sciences, Bishop’s University, Sherbrooke, Canada; 3 Département des Sciences Biologiques, Université du Québec à Montréal, Montréal, Canada

**Keywords:** Allocation trade-offs, Seasonal fluctuations, Oxidative damages, Rodents, Antioxidants, Food availability

## Abstract

Life-history allocation trade-offs are dynamic over time and space according to the ecological and demographical context. Fluctuations in food availability can affect physiological trade-offs like oxidative status regulation, reflecting the balance between pro-oxidant production and antioxidant capacity. Monitoring the spatio-temporal stability of oxidative status in natural settings may help understanding its importance in ecological and evolutionary processes. However, few studies have yet conducted such procedures in wild populations. Here, we monitored individual oxidative status in a wild eastern chipmunk (*Tamias striatus*) population across the 2017 summer active period and over three study sites. Oxidative damage (MDA: Malondialdehyde levels) and non-enzymatic antioxidant levels (FRAP: Ferric reducing antioxidant power and HASC: Hypochlorous acid shock capacity) were quantified across time and space using assays optimized for small blood volumes. Our results showed an increase in oxidative damage mirrored by a decrease in FRAP throughout the season. We also found different antioxidant levels among our three study sites for both markers. Our results also revealed the effects of sex and body mass on oxidative status. Early in the active season, females and individuals with a greater body mass had higher oxidative damage. Males had higher HASC levels than females throughout the summer. This study shows that oxidative status regulation is a dynamic process that requires a detailed spatial and temporal monitoring to yield a complete picture of possible trade-offs between pro-oxidant production and antioxidant capacity.

## Introduction

Most resources are limited and fluctuate through time and space in natural environments, which makes it difficult for living organisms to invest maximally in every adaptive trait. Trade-offs between resources allocated to different traits and the fitness consequences of these trade-offs are thus a central theme of life-history theories (Stearns, 1992; Van Noordwijk & De Jong, 1986). At the core of this trade-off dynamic is the potential for an individual to differentially acquire and allocate resources to self-maintenance or reproduction (Van Noordwijk & De Jong, 1986; Zera & Harshman, 2001).

Such variation can be driven by individual differences, as well as external factors. Indeed, different environmental conditions across habitats, either at the spatial or temporal scale, can impact resource intake or make some allocation strategies more successful than others. This in turn can lead to differences in fitness related traits such as survival or reproductive output (Alaux et al., 2017; Cooper & Kruuk, 2018; Rayor, 1985). For example, higher food availability generally allows greater simultaneous investment in traits requiring the same resources (Zera & Harshman, 2001), whereas lower food availability can divert resource allocation toward either short term fitness or survival (Ballinger, 1977; Erikstad et al., 1998; Kaitala, 1991; Zera & Harshman, 2001).

Recently, eco-physiological studies have highlighted that trade-offs can be detected at the physiological level, for example by examining oxidative status: the balance between pro-oxidants levels and cellular defense mechanisms (Birnie-Gauvin et al., 2017; Costantini, 2014; Monaghan & Costantini, 2014). Pro-oxidants, such as reactive species, can be generated as by products of the energetic metabolism. Their levels is thus expected to increase with energy expenditure or the expression of physiologically demanding processes, such as growth, increased reproductive effort or the activation of the immune response (Blount et al., 2016; Costantini & Møller, 2009; Smith, Nager & Costantini, 2016). Reactive species can damage various cellular components such as DNA, proteins and lipids, causing a state of oxidative stress if the cell’s protection and reparation mechanisms are outmatched (Halliwell & Gutteridge, 2015). The accumulation of oxidative damage could contribute to accelerate ageing and reduce survival, thus impairing long term fitness (Costantini & Dell’Omo, 2015; Finkel & Holbrook, 2000). Moreover, it has been shown that oxidative stress can be a physiological constraint and may shape the expression of life history traits (Cram et al., 2015; Smith, Nager & Costantini, 2016; Stier et al., 2012).

Among multiple physiological processes, oxidative damage can be prevented through the use of a wide array of exogenous (e.g., vitamins) and endogenous (e.g., enzymes) antioxidant molecules that neutralize reactive species before they can oxidize cellular components (Halliwell & Gutteridge, 2015). While exogenous antioxidants are generally acquired in the diet, endogenous antioxidants are produced by the cellular machinery (Halliwell & Gutteridge, 2015; Pamplona & Costantini, 2011). Several supplementation studies have shown that antioxidants can circumvent the constraint of oxidative stress on the expression of costly life history traits (Carbeck et al., 2018; Catoni, Peters & Schaefer, 2008; Costantini et al., 2016; De Ayala, Martinelli & Saino, 2006; Hall et al., 2010; Messina et al., 2017; Parolini et al., 2018). The synthesis of endogenous antioxidant compounds could however require energy and lead to a decreased availability of macronutrients for other proteins synthesis (Costantini, 2014; Lee et al., 2013; Li et al., 2014; Pamplona & Costantini, 2011). Thus, allocation trade-offs for antioxidant protection can represent another mechanism by which oxidative stress impacts life history traits (Costantini, 2014).

Oxidative status regulation can fluctuate according to external factors such as food availability. Increased food availability has been shown to increase antioxidant capacity and/or decrease oxidative damage (Costantini et al., 2018; Costantini et al., 2009; Fletcher et al., 2013; Van de Crommenacker et al., 2011). This could be due to an increase in exogenous antioxidant intake, an increase in endogenous antioxidant synthesis or lower foraging efforts (Beaulieu & Schaefer, 2013; Catoni, Peters & Schaefer, 2008; Lee et al., 2013; Li et al., 2014; Smith, Nager & Costantini, 2016; Van de Crommenacker et al., 2011). Additionally, changes in main feeding sources within a year can also affect the mechanisms regulating oxidative status (Catoni, Peters & Schaefer, 2008; Cohen, Mcgraw & Robinson, 2009). Fluctuations in resource availability are therefore likely to impact their allocation in oxidative status regulation (Costantini, 2014), as they do for energy allocation (Hoy et al., 2016; Perrin & Sibly, 1993).

Little is known about the determinants of among-individual variation in oxidative status in natural environments (Beaulieu & Costantini, 2014; Chainy, Paital & Dandapat, 2016). Oxidative status markers are generally moderately heritable and show a large environmental variance, probably because oxidative status regulation is tightly linked to the ecological and physiological context of living species (Kahar et al., 2016; Losdat et al., 2014; Norte et al., 2009). Indeed, several physiological adaptations have evolved to allow organisms to express life-history traits while coping with fluctuations in environment quality. For example, torpor, expressed in small mammals to minimize energy expenditure in unfavorable periods, is accompanied by fluctuations in oxygen levels during arousal cycles, which could cause oxidative stress and in turn be countered by increased endogenous antioxidant levels (Niu et al., 2018; Okamoto et al., 2006; Orr et al., 2009; Vucetic et al., 2013, but see Page et al., 2009). Following emergence from torpor, some organisms must restore their depleted body tissues and reserves, while they initiate reproduction (Michener, 1998; Siutz, Franceschini & Millesi, 2016). At the same time, they could rely mostly on exogenous antioxidants to regulate their oxidative status. Yet, such physiological trade-offs between key ecological trait expression and oxidative status regulation have rarely been monitored in natural settings.

Here we monitored the oxidative status in a wild eastern chipmunk (*Tamias striatus*) population on three study sites throughout their active season in 2017. We repeatedly sampled marked individuals after the emergence from torpor in the spring, in the middle of summer and during late-summer lactation, to assess the variations in oxidative status components. We simultaneously assayed malondialdehyde (MDA) levels, an index of oxidative damage, as well as the ferric reducing antioxidant power (FRAP) and the hypochlorous acid shock capacity (HASC), two total non-enzymatic antioxidant capacity markers. This allowed us to monitor fluctuations in antioxidant protection and the overall potential impact on tissue degradation, providing a more complete understanding of the oxidative status regulation (Costantini & Verhulst, 2009). We predict that trade-offs between oxidative status regulation and the expression of physiologically demanding traits can shift according to individual traits and environmental factors. For instance, torpor expression can induce muscle atrophy and its regeneration following emergence could lead to an indirect increase in reactive species production and oxidative damage (Amunugama et al., 2016; Wickler, Hoyt & Van Breukelen, 1991). Moreover, while increased oxidative damage is not necessarily paralleled by a decrease in antioxidant protection (Costantini & Verhulst, 2009), we could expect either no changes or a decrease in antioxidant levels, due to the non-enzymatic nature of the markers used. Similarly, a greater energy expenditure during lactation should increase oxidative stress through the same general mechanism. Furthermore, the trade-offs allocation dynamic should be affected by changes in food availability, which is possibly linked to energy and dietary antioxidant acquisition. Thus, increased food availability should favor a better antioxidant protection. Lastly, chipmunks that are spatially closer and have access to the same resources should display similar oxidative status.

## Material and Methods

### Study site and data collection

This study took place in 2017 and is part of a long-term monitoring of wild eastern chipmunks that started in 2005 in Southern Québec (Canada). Eastern chipmunks are ground dwelling solitary rodents that hoard seeds from masting trees in their burrows (Snyder, 1982). The large annual fluctuation in beech seeds production in the fall affects both above-ground activity and reproduction events in this species (Bergeron et al., 2011b; Munro, Thomas & Humphries, 2008). Chipmunks generally reproduce the summer preceding a beech masting event and the following spring, hence synchronizing juvenile emergence with high seed availability (Bergeron et al., 2011b). Non-mast years are characterized by extreme suppression of above-ground activity (Munro, Thomas & Humphries, 2008). On the northern part of their distribution, chipmunks spend the winter in torpor and rely on food reserves, whose quality and size can affect torpor pattern duration and intensity (Landry-Cuerrier et al., 2008; Munro, Thomas & Humphries, 2005).

In our study system, an American beech tree *(Fagus grandifolia*) mast occurred in 2017. Chipmunks reproduced in the summer and their juveniles emerged from their maternal burrow in the time of high food availability, in early fall (Bergeron et al., 2011b). Therefore, at the time of the study, the population was essentially composed of adults. Chipmunks were monitored at three sites (6.8 ha, 6.8 ha and 3.2 ha), where dominant seed producing tree species are the American beech, the sugar maple (*Acer saccharum*) and the red maple (*A. rubrum*) (see St-Hilaire, Réale & Garant, 2017 for details). Although chipmunks mostly target seeds, during periods of low seed abundance they also feed on herbaceous bulbs such as trout lily (*Erythronium americanum*) and spring beauty (*Claytonia caroliniana*). They also have been observed to eat fruits (black cherries, strawberries), invertebrates (snails, slugs, caterpillars) and mushrooms (Careau et al., 2013; Elliott, 1978; Humphries et al., 2002).

Individuals were monitored through mark and recapture using Longworth traps (Longworth Scientific Instruments, Abingdon, UK). Sites were systematically trapped once a week from May to August, from 0800 to dusk and traps were checked every 2 h. Every individual was sexed at the first capture and marked with a unique alphanumeric ear tag. Body mass was measured at each capture. The minimal known age of every new individual was determined following Bergeron et al. (2011a): new adults were considered to be 1 year old at the year of first capture.

### Blood collection

We collected blood during three sampling periods, at the end of May (spring), in June (summer), and in August (late summer) 2017. Sampling periods lasted from 8 to14 days and the three sites were trapped systematically to sample every active individual. Following Bergeron et al. (2011a), the blood was collected in up to four 70 µL heparinized glass capillary tubes after clipping a toenail. Blood capillaries were kept at 4 °C right after collection for no longer than 6 h. Then, they were centrifuged at 14 800 g for 6 min and plasma was stored at −15 °C before being transferred to −80 °C, within 6 days. This procedure was used for all three sampling periods. Samples were assayed 4 to 7 months after the first blood sampling period. Animals were captured and handled in compliance with the Canadian Council on Animal Care (#A2016-01 - Bishop’s University) and the Ministère des Ressources naturelles et de la Faune du Québec (#2017-05- 01-102-05-S-F). We collected 204 plasma samples on 96 individuals, representing 90% of the monitored population (25 chipmunks were sampled once, 34 twice and 37 three times). Sample sizes for each sex, period and sites are available in Table S1 in Supporting Information.

### Oxidative stress markers measurements

Plasma oxidative damage and antioxidant levels were assayed following Langille et al.’s (2018) methods, which are optimised for small blood volumes. We thus only provide a brief overview of these assays.

#### Oxidative damage

Oxidative damage were estimated with plasma total malondialdehyde (MDA, µmol L^−1^), a widely used lipid peroxidation biomarker (Beaulieu & Costantini, 2014), which we assayed using a high-performance liquid chromatography (HPLC). We added 2.5 µL of butylated hydroxytoluene (0.1 M) in anhydrous ethanol and 5 µL of NaOH (2.0 M) to a 20 µL aliquot of plasma or standard (1,1,3,3-tetraethoxypropane). After a 30 min incubation in a shaking dry block set at 60 °C, 100 µL of aqueous trichloroacetic acid (15% V/V) was added. The samples were placed in an ice bath for 5 min and spun 10 min at 14 000 g and 4 °C. To the supernatant (100 µL), 50 µL of 2-thiobarbituric acid (0.375% w/v) in HCl (0.25 M) was added, followed by a 60 min heating at 100 °C in a dry bath incubator. Samples were then cooled on ice and centrifuged 5 min at 14 000 g and 4 °C, before HPLC analysis. We assayed all blood samples in duplicate when the plasma volume was sufficient (*n* = 63 sampled once, and 89 sampled in duplicate) and samples with leftover plasma (*n* = 31) were assayed a third time. Intra and inter assay coefficients of variation were 10.86% and 16.01%, respectively.

#### Antioxidant protection

Total non-enzymatic antioxidant power was assessed with two markers: the ferric reducing/antioxidant power (FRAP) assay and the hypochlorous acid shock capacity (HASC) assay. These markers assay antioxidant power with contrasting mechanisms (see Langille et al., 2018 for details) and comparative studies have found no correlation or a weak positive correlation between them (Costantini, 2011; Vassalle et al., 2004). They are, therefore likely to depict different and complementary pictures of the antioxidant status in sampled individuals (Langille et al., 2018; Vassalle et al., 2004).

### The ferric reducing/antioxidant power (FRAP) assay

The FRAP assay is based on ferric to ferrous ion reduction by the plasma at low pH. It allows the colored ferrous-tripyridyltriazine complex to form, which can be quantified by spectrophotometry using mM FeSO_4_ equivalent (Benzie & Strain, 1996). This assay has been criticized for being highly sensitive to uric acid levels (Benzie & Strain, 1996; Cohen, Mcgraw & Robinson, 2009; Costantini, 2011), so here we used a uric acid independent FRAP assay. A 5 µL aliquot of plasma or standard (FeSO_4_, see Langille et al., 2018 for details) was mixed 1:1 with ultrapure water-containing uricase (1.00 U/mL) and incubated 5 min at room temperature on a 2D shaker. Then, 200 µL of FRAP reagent was added and absorbance was read at 593 nm using an iMark Filter absorbance microplate reader (Bio-Rad Laboratories Ltd., Mississauga, ON, Canada) after a 30 min incubation with shaking at room temperature. Blood samples were assayed in duplicate when the plasma volume was sufficient (*n* = 26 sampled once, and 120 sampled in duplicate) and samples with leftover plasma (*n* = 20) were assayed a third time. Intra and inter assay coefficients of variation were 4.78% and 9.12%, respectively.

#### The hypochlorous acid shock capacity (HASC) assay

The HASC assay is based upon the widely used Diacron International’s OXY-adsorbent test™ kit. It yields the total non-enzymatic antioxidant power through the amount of HOCl (mmol) required to fully oxidize a plasma sample. A 1 µL aliquot of plasma and 100 µL of oxidant solution (hypochlorous acid, see Langille et al., 2018 for details) were added to 100 µL of ultrapure water, followed by a 10 minutes incubation at room temperature on a 2D shaker. After that, 20 µL of N, N-dimethyl-p-phenylenediamine (83 µL in 50 mL anhydrous ethanol) was quickly added and absorbance was read at 515 nm after a 60 s incubation with shaking using the iMark Filter absorbance microplate reader. As for MDA and FRAP, blood samples were assayed in duplicate when possible (*n* = 21 sampled once, and 71 sampled in duplicate) and samples with leftover plasma (*n* = 13) were assayed a third time. Intra and inter assay coefficients of variation were 2.06% and 2.93%, respectively.

For each assay analysis, samples were randomized in terms of individual identification and sampling period to avoid any possible confounding effects caused by laboratory conditions.

### Statistical analyses

We ran linear mixed-effect models to assess the influence of individual and environmental variables on each marker using the R package lme4 (Bates et al., 2014). We used likelihood ratio tests (LRT) to compare increasing structure of complexity with assay number, chipmunk identity and sample unique identity nested in chipmunk identity as random effects. The full fixed effects model included environmental variables (sampling period and site), individual variables (sex, mass and minimal known age) and methodological variables (time spent in the trap before blood collection, blood collection duration, haemolysis, sample storage time at 4 °C and −15 °C). Additionally, for MDA, time spent in the HPLC queue was also included. All biologically relevant second-order interactions between environmental and individual states fixed effects were included in the models (sampling period × mass and sampling period × sex; full models are shown in Tables S2–S4).

We tested for within-sample correlations between markers by averaging replicates into one mean value for each marker. We removed hemolyzed samples (30 for MDA, 29 for FRAP and 16 for HASC) because population level analyses showed non-biological effect of haemolysis on MDA and FRAP. We then ran a linear mixed-effect model for each possible marker pair (i.e., MDA-FRAP, FRAP-HASC and HASC-MDA), in which the first marker of the pair was the response variable and the second a dependent variable. Significant fixed effects and second order interactions from the individual and environmental determinant analysis of each marker present in the models were included as covariates.

Moreover, we analysed the repeatability of the sampled markers with the R package rptR and estimated the variance components and confidence intervals (CI) from a linear mixed-effect models with chipmunk identity fitted as a random factor (Nakagawa & Schielzeth, 2010). Confidence intervals and standard errors were calculated from parametric bootstraps and permutations that created the distributions of likelihood ratios (set at 1000 times each). Repeatability was estimated on the whole dataset and on each possible sampling period pair (May–June, June–August and May–August).

We also analysed whether sampled individuals showed different reaction norms, for each marker, by testing the addition of Julian day within chipmunk ID slopes to the random effect structure used in populational determinant analyses, using LRT. Fixed effects included Julian day and each markers’ significant fixed effects from the populational determinant analyses (see Tables 1–3), except for the sampling period.

**Table 1:** Final model of determinants of population MDA levels (oxidative damage) obtained from a linear mixed-model and simplified by a backward selection procedure. Female was the sex of reference, and May was the sampling period of reference. The model included 183 plasma samples from 92 chipmunks, all processed in 12 assay runs.

Components	Values	% of variance	LRT	*P* value
Chipmunk ID	0.66	14.0	37.50	<0.001
Sample unique ID	1.41	30.0	23.82	<0.001
Assay run	0.64	13.6	14.75	<0.001
Residual variance	1.99	42.3		
Variables	Coefficients	Std. Error	t value	*P* value
Intercept	0.91	2.98	0.30	0.762
Mass	0.10	0.04	2.59	0.010
Sex (male)	−1.92	0.57	3.36	0.001
Sampling period (June)	8.32	4.13	2.01	0.047
Sampling period (August)	10.03	5.08	1.98	0.051
Haemolysis	0.99	0.42	2.39	0.018
Sampling period (June) × Sex (male)	1.76	0.67	2.62	0.010
Sampling period (August) × Sex (male)	2.27	0.76	3.01	0.003
Sampling period (June) × Mass	−0.11	0.05	2.18	0.032
Sampling period (August) × Mass	−0.12	0.06	2.06	0.042

**Table 2:** Final model of determinants of population FRAP values (total non-enzymatic antioxidant power) obtained from a linear mixed-model and simplified by a backward selection procedure. Site 1 was the site of reference and May was the sampling period of reference. The model included 166 plasma samples from 83 chipmunks, all processed in eight assay runs.

Components	Values	% of variance	LRT	*P* value
Chipmunk ID	0.0016	8.7	46.48	<0.001
Sample unique ID	0.0087	46.7	190.53	<0.001
Assay run	0.0069	37.0	30.48	<0.001
Residual variance	0.0014	7.6		
Variables	Coefficients	Std. Error	t value	*P* value
Intercept	0.5638	0.0356	15.85	<0.001
Site (2)	0.0465	0.0212	2.19	0.032
Site (3)	0.0065	0.0233	0.28	0.781
Sampling period (June)	−0.0536	0.0194	2.76	0.007
Sampling period (August)	−0.0992	0.0236	4.20	<0.001
Hours stored at −15 °C	−0.0009	0.0003	3.16	0.002
Haemolysis	0.1134	0.0230	4.92	<0.001

**Table 3:** Final model of determinants of population HASC values (total non-enzymatic antioxidant power) obtained from a linear mixed-model and simplified by a backward selection procedure. Site 1 was the site of reference and females were the sex of reference. The model included 105 plasma samples from 73 chipmunks, all processed in 4 assay runs.

Components	Values	% of variance	LRT	*P* value
Chipmunk ID	0.0	0.0	1.56	0.211
Sample unique ID	0.0000038	22.8	33.02	<0.001
Assay run	0.0000059	35.3	24.21	<0.001
Residual variance	0.0000070	41.9		
Variables	Coefficients	Std. Error	t value	*P* value
Intercept	0.08766	0.00132	66.52	<0.001
Site (2)	0.00137	0.00062	2.19	0.031
Site (3)	−0.00127	0.00074	1.73	0.088
Sex (male)	0.00169	0.00054	3.11	0.003

Finally, we also ran a mixed linear model to analyse 2017 populational spring mass gain. Mass measurements from early May to June 15th were considered (based on our personal observations that mid June is the end of the spring mass gain period). Chipmunk identity was included as a random effect and full model fixed effects structure included Julian day as a linear effect, Julian day as a quadratic effect, sex and the interaction between sex and Julian day.

All analyses were performed using the software R 3.4.2 (R Core Development Team R, 2016). The significance of the fixed effects was estimated with the package lmerTest (Kuznetsova, Brockhoff & Christensen, 2017). Final models were determined by sequentially removing the least significant term from the model based on its *P*-value (*α* = 0.05) and comparing with a LRT this new model to the previous one, until all remaining variables were significant (Crawley, 2012).

## Results

### Relationships between markers

The three oxidative status variables were not significantly associated with each other within a blood sample (MDA-FRAP: *β* =  − 1.373 ± 1.547, *P* = 0.377, *n* = 142; FRAP-HASC: *β* = 3.427 ± 2.915, *P* = 0.243, *n* = 90; MDA-HASC: *β* =  − 0.00012 ± 0.00019, *P* = 0.530, *n* = 93).

### Individual and environmental determinants at the population level

#### MDA

**Figure 1:**
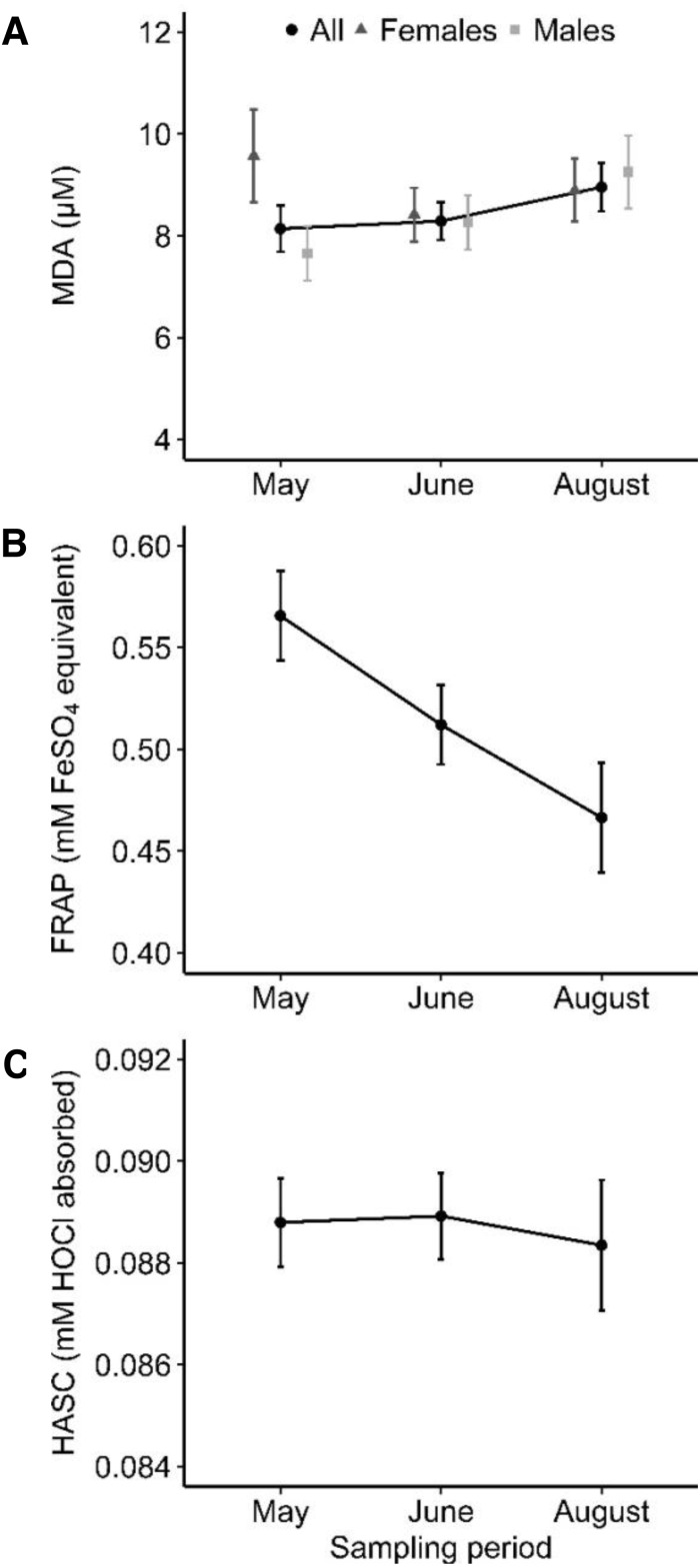
Population-level values of oxidative stress markers at each sampling periods (with 95% upper and lower confidence intervals). (A) The predicted values for MDA (oxidative damage) were obtained from the final model presented in Table 1. The solid line represents the populational trend (*n* = 183 plasma samples from 92 eastern chipmunks). (B) The predicted values for FRAP (total antioxidant capacity) were obtained from the final model presented in Table 2 (*n* = 166 plasma samples from 83 chipmunks). (C) The predicted values for HASC (total antioxidant capacity) were obtained from the final model presented in Table 3 (*n* = 105 plasma samples from 73 chipmunks).

**Figure 2:**
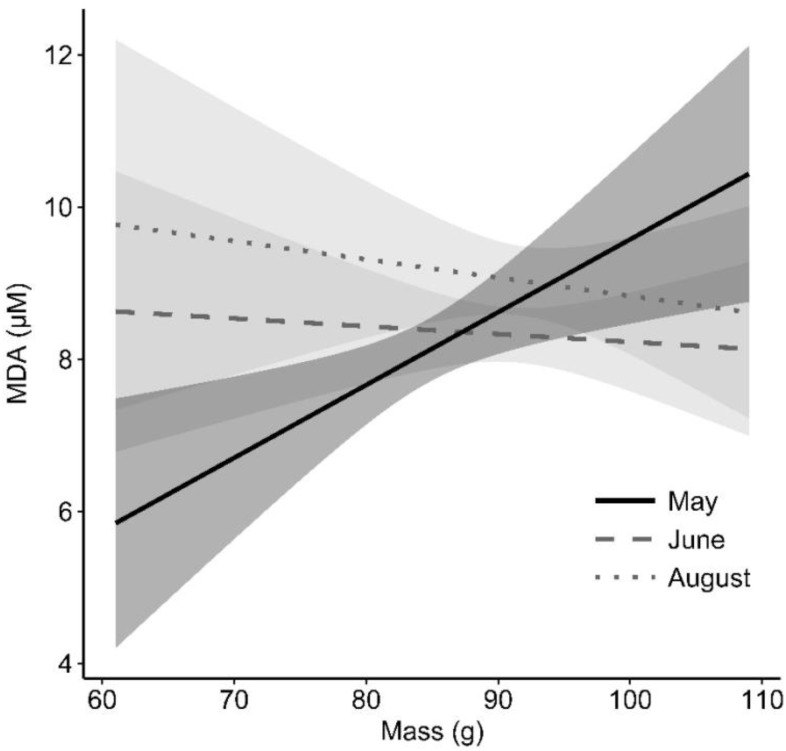
Effect of mass on MDA (oxidative damage) levels for each sampling period in an eastern chipmunk population. May: *n* = 71; June: *n* = 73; August: *n* = 39 plasma samples. The predicted values (lines) for MDA were obtained from the final model presented in Table 1. The grey areas represent the upper and lower 95% confidence intervals.

Overall, MDA level increased by 10% over the course of the summer (Fig. 1A). Our analysis also revealed a significant sex by sampling period interaction (Table 1, Fig. 1A). Average female MDA level was higher than males in May and remained relatively stable throughout the summer, whereas average male MDA level increased over time (Fig. 1A). A significant interaction was also found between MDA and mass, which was mainly due to the increasing MDA level with mass in May and the absence of relationship between them during the two other periods (Table 1, Fig. 2). Age was not significantly related to MDA. Haemolysis of blood was related to increased MDA levels (Table 1). Assay number, chipmunk and plasma sample identities explained 57.7% of the total variance in MDA levels (Table 1). The full model is presented in Table S2.

#### FRAP

**Figure 3:**
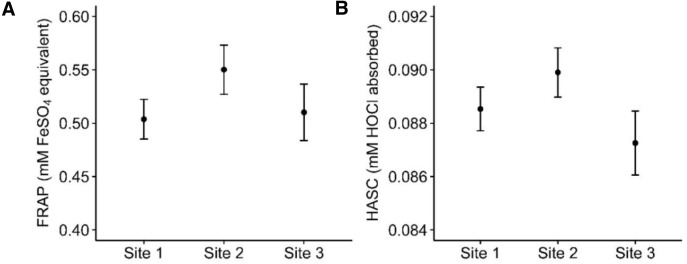
Total antioxidant capacity differences among sites in an eastern chipmunk population, in 2017, with upper and lower 95% confidence interval (error bars). (A) The predicted values for FRAP were obtained from the final model presented in Table 2 (Site1: *n* = 74; Site 2: *n* = 55; Site 3: *n* = 37 plasma samples). (B) The predicted values for HASC were obtained from the final model presented in Table 3 (Site1: *n* = 46; Site 2: *n* = 38; Site 3: *n* = 21 plasma samples).

Populational FRAP levels decreased by 18% throughout the sampling periods (Table 2, Fig. 1B). FRAP levels were also significantly higher at site 2 than site 1, although they both did not significantly differ from site 3 (Table 2, Fig. 3A). Mass and age were not significantly related to FRAP levels. Haemolysis and storage at −15 °C were related to increased and decreased FRAP values, respectively (Table 2). Assay number, chipmunk and plasma sample identities explained most of the total variance in FRAP levels (Table 2). The full model is presented in Table S3.

#### HASC

HASC values on site 2 were significantly higher than site 3, and marginally non-significantly higher than site 1 (Table 3, Fig. 3B). Males had higher HASC values than females (Table 3). Age, mass and sampling period as well as chipmunk handling and plasma storage conditions were not related to HASC values. Chipmunk identity did not explain a significant part of the variance but was kept in the model because the nested plasma sample identity was significant. Assay number also explained a significant part of the variance (Table 3). The full model is presented in Table S4.

### Repeatability estimates

Over the whole summer, we found a repeatability of 0.48 for MDA (95% CI [0.35–0.58]), 0.39 for FRAP (95% CI [0.28–0.50]) and 0.40 for HASC (95% CI [0.22–0.54]) among sampled individuals. Repeatability values and CI for each season pairs are presented in Table 4.

**Table 4:** Individual repeatability (r) estimates among sampling periods for oxidative damage (MDA) and antioxidant levels (FRAP and HASC). Variance components and confidence intervals (CI) were calculated from a linear mixed-effect models with chipmunk identity fitted as a random factor. CIs were calculated from parametric bootstraps and permutations, set at 1,000 times each.

Dataset		MDA		FRAP		HASC
	r	CI 95%	r	CI 95%	r	CI 95%
All sampling periods	0.48	[0.35–0.58]	0.39	[0.27–0.50]	0.40	[0.23–0.54]
May & June	0.66	[0.54–0.75]	0.57	[0.45–0.67]	0.50	[0.30–0.65]
June & August	0.53	[0.38–0.64]	0.54	[0.39–0.66]	0.62	[0.44–0.75]
May & August	0.38	[0.19–0.53]	0.59	[0.45–0.71]	0.40	[0.18–0.58]

### Individual reaction norm

The addition of individual random slopes within Julian day significantly improved model fitting for FRAP (*p* = 0.038, Table 5) but not for MDA (*p* = 0.875) nor HASC (*p* = 0.759).

**Table 5:** Random regression analyses of the effect of the Julian day on markers values. Addition of Julian day within chipmunk ID was compared to the random effect structure used in populational determinant analyses (Tables 1–3) using LRT. Fixed effects included Julian day and each markers’ significant fixed effects from the populational determinant analyses (Tables 1–3), except for the sampling period.

Marker	Random effect structure	Loglikelihood	D.f.	LRT	*P* value
MDA	Chipmunk ID	−735.73	11		
	Chipmunk ID × Julian day	−735.60	13	0.27	0.875
FRAP	Chipmunk ID	420.98	10		
	Chipmunk ID × Julian day	424.24	12	6.52	0.038
HASC	Chipmunk ID	978.88	9		
	Chipmunk ID × Julian day	979.16	11	0.55	0.759

### Populational spring mass

Populational mass levels increased during the analysed timeframe, although the quadratic effect slightly reduced the slope as Julian days increased (Table 6). Males weighed roughly 8.8 g more than females, however both sexes seemed to follow the same mass gain trajectory. Chipmunk identity explained 78.46% of the total variance in mass (Table 6). The full model is presented in Table S5.

**Table 6:** Final model of determinants of 2017 spring mass catch-up growth. The model was simplified using backward selection. Females were the sex of reference. The model included 540 mass measurements from May first to June 15th on 52 females and 70 males.

Components	Values	% of variance	LRT	*P* value
ID	33.79	78.46	515.50	<0.001
Residual variance	9.30	21.58		
Variables	Coefficients	Std. Error	*t* value	*P* value
Intercept	80.91	0.94	85.84	<0.001
Julian day (std)	3.40	0.17	20.24	<0.001
Julian day^2^ (std)	−0.35	0.14	2.41	0.016
Sex (male)	8.80	1.27	6.94	<0.001

## Discussion

The aim of this study was to assess the extent of spatio-temporal variation in the oxidative status of a wild eastern chipmunk population. We observed an increase in MDA, an index of oxidative damage, throughout the active season. This increase was mirrored by a decrease in FRAP, an index of total antioxidant power. Antioxidant levels measured with both FRAP and HASC were different among sites. MDA levels increased with body mass after torpor emergence, but that relationship disappeared during the following sampling periods. Moreover, females had higher oxidative damage than males at torpor emergence, whereas males had overall higher antioxidant HASC levels. These results provide a good example of the ecological and environmental contexts fluctuations that wild animals can experience within a year and their potential impact on allocation trade-offs and oxidative status regulation.

### Temporal variation at the population level

The monitoring of the oxidative status revealed a populational decrease in FRAP levels combined with an increase in MDA across the sampling periods. Our results are concordant with a previous study on eastern chipmunks within the same area that reported a temporal increase of free MDA levels during a mast year (Bergeron et al., 2011a). Chipmunks population dynamic is highly affected by temporal fluctuations in food availability, which is likely to affect dietary antioxidant acquisition and resource availability for antioxidant production (Bergeron et al., 2011b; Costantini, 2014; Isaksson, Sheldon & Uller, 2011). During mast years, seed availability is low in early summer, therefore chipmunks consume and hoard abundant herbaceous plant bulbs (Elliott, 1978). They then shift to hoarding red maple seeds in mid-summer as the spring plant disappear and the red maple seeds start falling (Elliott, 1978). During late summer, as in previous mast years, red maple seed depletion resulted in a decrease in food availability and reduced above ground activity, which lasted until beech seed masting in early fall (LaZerte & Kramer, 2016). Thus, a general decrease in food availability may be linked with a shift in oxidative status regulation observed in the studied adult population.

Non-mutually exclusive hypotheses can explain the differences in oxidative status observed between May and June when food was available. The shift between bulbs and seeds as the main feeding source may have changed the dietary antioxidant intake or the availability of macronutrients required for antioxidant synthesis. Both factors can impact the overall antioxidant levels (Cohen, Mcgraw & Robinson, 2009; Lee et al., 2013; Li et al., 2014). Also, the second sampling period in June occurred during the summer mating period. Mating activity can result in decreased time spent foraging and increased rate of aggressions between conspecifics (Buck & Barnes, 1999; Elliott, 1978; Tobler, Schlupp & Plath, 2011). These changes in behaviour could impair oxidative status regulation (Garratt et al., 2012; Rammal, Bouayed & Soulimani, 2010).

Moreover, we found that values obtained for of all three markers were repeatable over time (Table 4), suggesting a certain level of stability in oxidative status regulation for individuals. A closer inspection of our data revealed individual differences in reaction norms of oxidative status regulation across the sampling periods. There was significant among individual’s variation in FRAP levels through time but not for the other markers. However, a low statistical power may have impeded correct estimation of parameters (Martin et al., 2011). Future studies should, therefore, further explore individual differences in reaction norms for these markers using a larger dataset.

### Spatial variation in antioxidant capacity

We found that oxidative status regulation varied at a fine spatial scale, as shown by different antioxidant levels between the study sites for both markers. The overall trend suggests that chipmunks present at site 2 had higher antioxidant levels than the other sites (Fig. 3). In our study system, sites 1 and 2 are geographically close to each other (1 km) and located in a deciduous forest, whereas site 3 is located further (about 5 km) in a mixed forest (see St-Hilaire, Réale & Garant, 2017 for details). Therefore, we expected the latter to be the most different, given the close relationship between deciduous trees seed production, population dynamic and physiology of chipmunks (Bergeron et al., 2011b; Landry-Cuerrier et al., 2008). One explanation for the spatial difference in antioxidant levels observed might come from the among-site differences in herbaceous spring plant bulb density. As part of the long-term study, the density of herbaceous spring plants (Trout lily and American beauty) within a 10-meter radius of the occupied burrows was estimated and an additional analysis revealed that site 2 presented higher densities of herbaceous plants (3.59/m^2^ for Site 1, 7.45/m^2^ for Site 2 and 4.11/m^2^ for Site 3, *P*< 0.001, Table S6). During masting years, when seed availability is initially low, spring plant bulbs are hoarded and consumed by chipmunks (Elliott, 1978). Therefore, chipmunks with better access to spring plant bulbs may have had access to more dietary antioxidants or macronutrients. Oxidative damage, however, remained unchanged across sites possibly because chipmunks allocated resources to other traits of functions instead of minimizing oxidative damage, as observed in other studies. For instance, supplementation studies showed that extra resources available for juveniles were used to maximise growth rates, sometimes at the cost of oxidative status regulation (Costantini et al., 2018; Giordano, Costantini & Tschirren, 2015; Hall et al., 2010).

### Time-dependent mass effect on oxidative damage

There was a positive relationship between MDA levels and body mass during the late-May sampling period (Fig. 2). One possible explanation for this trend could lie in compensatory mass gain following torpor emergence. Muscle catabolism and atrophy can occur during hibernation, leading to considerable weight loss in many species including chipmunks (Levesque & Tattersall, 2010). A restoration period is therefore required after torpor suppression (Humphries, Thomas & Kramer, 2003). It appeared to be still ongoing during our first blood sampling period in late May, as shown by the temporal increase of mean populational mass (see Table 6). The high cellular activity caused by tissue generation is likely to favor reactive species production and result in oxidative stress, either because of an increased biosynthesis rate (Amunugama et al., 2016) or an increased metabolic rate (Halliwell & Gutteridge, 2015). In a wider allocation trade-off context, it is possible that some individuals favored compensatory mass gain over oxidative status regulation to maximize fitness returns at the following reproduction.

### Sex difference in oxidative status

After torpor emergence, females generally had higher oxidative damage than males (Fig. 1). As for other hibernating species (Buck & Barnes, 1999; Michener, 1992; Siutz et al., 2018), male eastern chipmunks cease torpor several days earlier than females and stay in a normothermic state before emergence (Dammhahn et al., 2017; Munro, Thomas & Humphries, 2005). This is likely to facilitate testicular recrudescence and initiate spermatogenesis before summer reproduction (Humphries, Thomas & Kramer, 2003; Siutz et al., 2018). During this pre-emergence euthermic period, males start eating their remaining cached food (Munro, Thomas & Humphries, 2005) hence possibly mitigating the oxidative costs of the catch-up phase by extending its duration. However, a closer analysis of spring populational mass did not show different mass gain rates between males than females (Table S5). Yet, females may not spend as much time in euthermia before emergence, possibly to benefit from conserving their food reserves for future gestation and lactation (Munro, Thomas & Humphries, 2005), which can occur during a low food availability period (Elliott, 1978; LaZerte & Kramer, 2016; Munro, Thomas & Humphries, 2005). Females, therefore, possibly experienced a shorter restoration period compared to males, which may have impaired oxidative status regulation. It may also explain why males had higher HASC levels across all summer (Table 3). However, this hypothesis remains to be tested experimentally.

Finally, we had little evidence for an oxidative cost of late-peak lactation by females, which occurred during our last sampling period. A previous study in this population reported an increase in oxidative damage with litter size (Bergeron et al., 2011a), and thus we expected to find greater MDA levels in females than males. Yet, even if all but three sampled females reproduced in 2017, their oxidative damage levels and FRAP levels in late summer were similar to males. Indeed, reproduction does not appear to be directly linked to oxidative damage. For example, recent meta-analyses have found no differences in oxidative status between sexes in mammals and a decrease in oxidative damage for breeding females when compared to non-breeding females (Blount et al., 2016; Costantini, 2018). It has therefore been suggested by various authors that physiological mechanisms can operate to protect either mothers or offspring from possible oxidative costs of reproduction (Alonso-Álvarez, Canelo & Romero-Haro, 2016; Blount et al., 2016; Costantini, 2018). Such physiological adaptations could be responsible for the observed similarities between lactating females and males in our study system, although this would have to be tested experimentally. Still, HASC levels were higher for males throughout all three sampling periods, suggesting that females may have experienced a greater oxidative challenge despite comparable MDA levels. Additionally to physiological adaptations, and as suggested in the present study, environmental driven changes could also impact oxidative status regulation (Catoni, Peters & Schaefer, 2008; Fletcher et al., 2013; Van de Crommenacker et al., 2011). Follow-up studies should, therefore, sample oxidative stress variation in a representative set of environmental conditions, including a non-masting year in this study system.

## Conclusions

Our study shows that oxidative status regulation is a dynamic process that requires a detailed spatial and temporal monitoring to provide a complete picture of possible trade-offs impacting its regulation in wild populations. We observed a populational decrease in antioxidant levels over the summer mirrored by an increased in oxidative damage, as well as different antioxidant levels among study sites that were possibly linked to food availability. Moreover, following torpor emergence, females and individuals with a faster catch-up mass gain had higher oxidative damage levels, two relationships that disappeared in the following sampling periods, when populational body mass reached a plateau. Our results, therefore, highlight that these trade-offs appear to be driven by environmental quality as well as physiology. Future challenges will include studying oxidative status in relation with fitness. This will be crucial to address the role of oxidative stress in shaping the expression and evolution of life-history traits.

##  Supplemental Information

10.7717/peerj.7801/supp-1 Datset S1 Raw data for oxydative status markers SampleID ID of the blood test (there are several blood tests by chipmunks)
Marker The marker name
Value The value of the replicate (average of injections of the same replicate for MDA)
Run The plate number (or lab day for MDA) in which the sample was located
Duplicate For MDA only, duplicate ID (aliquots of the same blood sample that were prepared in parallel)
Sit Time For MDA only, Time spent at room temperature in the HPLC queue
Variance Variance between measurements of the same blood sample, for a given marker
Hemo Hemolysis level of the plasma aliquot that generated the value (0 or 1). 1 means strong hemolysis. Note: This was not done for OXY, as the tubes were made almost empty and it was difficult to see the color of the plasma Since OXY was done overwhelmingly on remnants of other markers, the values for OXY were inferred from the other markers
ID Screen identity
Season Sampling period (P = end of May, E = end of June, A = end of August)
Jjul Julian day of the blood test (1 = January 1st)
Site Site Site on which the chipmunk was captured
Sex Sex of the tamia
Mass Net weight in grams of the chipmunk, just before the blood test
AgeMin Known minimum age (adult chipmunks caught are considered one year old when first caught, juveniles are ”zero” year old)
Botfly Number of botfly in the chipmunk at the time of blood sampling
BleedMinutes Time in minutes for the blood test (the stopwatch starts when the claw is cut, but keep in mind that you still had to take the time to identify the front chipmunk)
TrapHours Estimate of the time spent in the trap, in hours, before the blood test (approximate since the traps are visited every 2 hours)
4DegreesHours Time, in hours, that the blood passed at 4 degrees before being centrifuged and frozen (the target was ¡5h, some were exceeded)
Minus15Hours Time in hours spent at -15 degrees before transfer to -80

10.7717/peerj.7801/supp-2 Table S1 Summary of the 2017 sample sizes for each sampling period and site (Females, Males)

10.7717/peerj.7801/supp-3 Table S2 Full model of determinants of population MDA levels (oxidative damage) obtained from a linear mixed-model simplified by a backward selection Female was the sex of reference and May was the sampling period. The model included 183 plasma samples from 92 chipmunks, all processed in 12 assay runs.

10.7717/peerj.7801/supp-4 Table S3 Full model of determinants of population FRAP values (total non-enzymatic antioxidant power) obtained from a linear mixed-model simplified by a backward selection Site 1 was the site of reference and May was the sampling period of reference. The model included 166 plasma samples from 83 chipmunks, all processed in 8 assay runs.

10.7717/peerj.7801/supp-5 Table S4 Full model of determinants of population HASC values (total non-enzymatic antioxidant power) obtained from a linear mixed-model simplified by a backward selection Site 1 was the site of reference and females were the sex of reference. The model included 105 plasma samples from 73 chipmunks, all processed in 4 assay runs.

10.7717/peerj.7801/supp-6 Table S5 Full model of determinants of 2017 spring mass catch-up growth The model was further simplified using backward selection. Females were the sex of reference. The model included 540 mass measurements from May first to June 15th on 52 females and 70 males.

10.7717/peerj.7801/supp-7 Table S6 Edible spring plant density differences among sites, tested using a linear model Site 1 was the site of reference. Site estimations are based on microhabitat sampling of burrows (Site 1: *n* = 58, Site 2: *n* = 33, Site 3: *n* = 11).

## Additional Information and Declarations

### Field Study Permissions

The following information was supplied relating to field study approvals (i.e., approving body and any reference numbers): The Ministère des Ressources naturelles et de la Faune du Québec SEG provided permit #2017-05- 01-102-05-S-F. Nature Conservancy of Canada allowed us to conduct this research on their lands.

### Data Availability

The following information was supplied regarding data availability: The raw data are available as a Supplemental File.
