# Supplementary material for: Spatio-temporal variation in oxidative status regulation in a small mammal
Source: PeerJ. 2019 Oct 8;7:e7801. doi: 10.7717/peerj.7801 (PMC6788435; doi:10.7717/peerj.7801)
Supplement: Table S1 [file peerj-07-7801-s002.docx]

| Sampling period | Site 1 | Site 2 | Site 3 |
| --- | --- | --- | --- |
| May | 14, 17 | 12, 14 | 7, 10 |
| June | 14, 19 | 17,14 | 5, 10 |
| August | 11, 13 | 9, 5 | 6, 7 |
