# Supplementary material for: Spatio-temporal variation in oxidative status regulation in a small mammal
Source: PeerJ. 2019 Oct 8;7:e7801. doi: 10.7717/peerj.7801 (PMC6788435; doi:10.7717/peerj.7801)
Supplement: Table S2 — Female was the sex of reference and May was the sampling period. The model included 183 plasma samples from 92 chipmunks, all processed in 12 assay runs. [file peerj-07-7801-s003.docx]

| Components | Values | % of variance | LRT | *P* value |
| --- | --- | --- | --- | --- |
| Chipmunk ID | 0.66 | 14.0 | 37.16 | <0.001 |
| Sample unique ID | 1.41 | 30.0 | 23.81 | <0.001 |
| Assay run | 0.64 | 13.6 | 14.87 | <0.001 |
| Residual variance | 1.99 | 42.3 |  |  |
| Variables | Coefficients | Std. Error | t value | *P* value |
| Intercept | 1.55 | 3.13 | 0.50 | 0.620 |
| Hours in trap before sampling | -0.15 | 0.24 | 0.61 | 0.542 |
| Sampling duration in minutes | 0.06 | 0.04 | 1.52 | 0.128 |
| Hours stored at 4°C | -0.24 | 0.15 | 1.56 | 0.120 |
| Hours stored at -20°C | 0.00 | 0.00 | 0.38 | 0.706 |
| Hours in HPLC queue | -0.01 | 0.03 | 0.48 | 0.637 |
| Haemolysis | 0.99 | 0.42 | 2.39 | 0.018 |
| Site (2) | 0.09 | 0.38 | 0.24 | 0.812 |
| Site (3) | -0.05 | 0.42 | 0.11 | 0.912 |
| Age | 0.05 | 0.13 | 0.38 | 0.703 |
| Mass | 0.10 | 0.04 | 2.59 | 0.010 |
| Sex (male) | -1.92 | 0.57 | 3.36 | <0.001 |
| Sampling period (June) | 8.32 | 4.13 | 2.01 | 0.047 |
| Sampling period (August) | 10.03 | 5.08 | 1.96 | 0.051 |
| Sampling period (June) x Sex (male) | 1.76 | 0.67 | 2.65 | 0.010 |
| Sampling period (August) x Sex (male) | 2.27 | 0.76 | 3.01 | 0.003 |
| Sampling period (June) x Mass | -0.11 | 0.05 | 2.18 | 0.032 |
| Sampling period (August) x Mass | -0.12 | 0.06 | 2.06 | 0.042 |
