# Supplementary material for: Spatio-temporal variation in oxidative status regulation in a small mammal
Source: PeerJ. 2019 Oct 8;7:e7801. doi: 10.7717/peerj.7801 (PMC6788435; doi:10.7717/peerj.7801)
Supplement: Table S3 — Site 1 was the site of reference and May was the sampling period of reference. The model included 166 plasma samples from 83 chipmunks, all processed in 8 assay runs. [file peerj-07-7801-s004.docx]

| Components | Values | % of variance | LRT | *P* value |
| --- | --- | --- | --- | --- |
| Chipmunk ID | 0.0016 | 8.7 | 46.48 | <0.001 |
| Sample unique ID | 0.0087 | 46.7 | 190.53 | <0.001 |
| Assay run | 0.0069 | 37.0 | 30.48 | <0.001 |
| Residual variance | 0.0014 | 7.6 |  |  |
| Variables | Coefficients | Std. Error | t value | *P* value |
| Intercept | 0.7527 | 0.2059 | 3.66 | <0.001 |
| Hours in trap before sampling | -0.0045 | 0.0147 | 0.31 | 0.758 |
| Sampling duration in minutes | 0.0028 | 0.0023 | 1.24 | 0.216 |
| Hours stored at 4°C | 0.0058 | 0.0100 | 0.58 | 0.563 |
| Hours stored at -15°C | -0.0009 | 0.0003 | 3.16 | 0.002 |
| Haemolysis | 0.1134 | 0.0230 | 4.92 | <0.001 |
| Site (2) | 0.0465 | 0.0212 | 2.19 | 0.032 |
| Site (3) | 0.0065 | 0.0233 | 0.28 | 0.781 |
| Minimal known age | 0.0045 | 0.0082 | 0.55 | 0.581 |
| Mass | -0.0011 | 0.0013 | 0.81 | 0.421 |
| Sex (male) | 0.0005 | 0.0207 | 0.02 | 0.982 |
| Sampling period (June) | -0.0536 | 0.0194 | 2.76 | 0.007 |
| Sampling period (August) | -0.0992 | 0.0236 | 4.20 | <0.001 |
| Sampling period (June) x Sex (male) | -0.0075 | 0.0371 | 0.20 | 0.841 |
| Sampling period (August) x Sex (male) | -0.0369 | 0.0475 | 0.78 | 0.439 |
| Sampling period (June) x Mass | 0.0021 | 0.0033 | 0.64 | 0.523 |
| Sampling period (August) x Mass | 0.0019 | 0.0039 | 0.48 | 0.630 |
