# Supplementary material for: Spatio-temporal variation in oxidative status regulation in a small mammal
Source: PeerJ. 2019 Oct 8;7:e7801. doi: 10.7717/peerj.7801 (PMC6788435; doi:10.7717/peerj.7801)
Supplement: Table S4 — Site 1 was the site of reference and females were the sex of reference. The model included 105 plasma samples from 73 chipmunks, all processed in 4 assay runs. [file peerj-07-7801-s005.docx]

| Components | Values | % of variance | LRT | *P* value |
| --- | --- | --- | --- | --- |
| Chipmunk ID | 0.0 | 0.0 | 1.56 | 0.211 |
| Sample unique ID | 0.0000038 | 22.7 | 33.08 | <0.001 |
| Assay run | 0.0000059 | 35.4 | 24.16 | <0.001 |
| Residual variance | 0.0000070 | 41.9 |  |  |
| Variables | Coefficients | Std. Error | t value | *P* value |
| Intercept | 0.09094 | 0.00693 | 13.13 | <0.01 |
| Hours in trap before sampling | 0.00034 | 0.00051 | 0.67 | 0.507 |
| Sampling duration in minutes | 0.00014 | 0.00007 | 1.87 | 0.065 |
| Hours stored at 4°C | -0.00008 | 0.00031 | 0.26 | 0.798 |
| Hours stored at -15°C | -0.00001 | 0.00001 | 1.46 | 0.149 |
| Haemolysis | 0.00005 | 0.00086 | 0.06 | 0.953 |
| Site (2) | 0.00137 | 0.00062 | 2.19 | 0.031 |
| Site (3) | -0.00127 | 0.00074 | 1.73 | 0.088 |
| Minimal known age | 0.00016 | 0.00026 | 0.61 | 0.547 |
| Mass | 0.00001 | 0.00005 | 0.28 | 0.780 |
| Sex (male) | 0.00169 | 0.00054 | 3.11 | 0.003 |
| Sampling period (June) | 0.00002 | 0.00065 | 0.03 | 0.976 |
| Sampling period (August) | -0.00033 | 0.00084 | 0.39 | 0.698 |
| Sampling period (June) x Sex (male) | -0.00105 | 0.00164 | 0.64 | 0.522 |
| Sampling period (August) x Sex (male) | -0.00005 | 0.00191 | 0.03 | 0.980 |
| Sampling period (June) x Mass | 0.00011 | 0.00010 | 1.07 | 0.287 |
| Sampling period (August) x Mass | 0.00007 | 0.00012 | 0.56 | 0.576 |
