# Supplementary material for: Spatio-temporal variation in oxidative status regulation in a small mammal
Source: PeerJ. 2019 Oct 8;7:e7801. doi: 10.7717/peerj.7801 (PMC6788435; doi:10.7717/peerj.7801)
Supplement: Table S5 — The model was further simplified using backward selection. Females were the sex of reference. The model included 540 mass measurements from May first to June 15th on 52 females and 70 males. [file peerj-07-7801-s006.docx]

| Components | Values | % of variance | LRT | *P* value |
| --- | --- | --- | --- | --- |
| ID | 33.78 | 78.42 | 515.50 | < 0.001 |
| Residual variance | 9.30 | 21.58 |  |  |
| Variables | Coefficients | Std. Error | t value | P value |
| Intercept | 80.91 | 0.94 | 85.84 | < 0.001 |
| Julian day (std) | 3.40 | 0.17 | 20.24 | < 0.001 |
| Julian day² (std) | -0.35 | 0.14 | 2.41 | 0.016 |
| Sex (male) | 8.80 | 1.27 | 6.94 | < 0.001 |
| Julian day (std): Sex (male) | 0.05 | 0.31 | 0.16 | 0.871 |
