# Supplementary material for: Spatio-temporal variation in oxidative status regulation in a small mammal
Source: PeerJ. 2019 Oct 8;7:e7801. doi: 10.7717/peerj.7801 (PMC6788435; doi:10.7717/peerj.7801)
Supplement: Table S6 — Site 1 was the site of reference. Site estimations are based on microhabitat sampling of burrows (Site 1: n = 58, Site 2: n = 33, Site 3: n = 11). [file peerj-07-7801-s007.docx]

| Variables | Coefficients | Std. Error | t value | *P* value |
| --- | --- | --- | --- | --- |
| Intercept | 3.5914 | 0.3201 | 11.221 | < 0.001 |
| Site (2) | 3.8632 | 0.5315 | 7.268 | < 0.001 |
| Site (3) | 0.5177 | 0.8016 | 0.646 | 0.520 |
